# The case for standardising robotic curriculum globally

**DOI:** 10.52054/FVVO.14.2.022

**Published:** 2022-07-01

**Authors:** G Moawad, S Rahman, E Saridogan

**Affiliations:** Obstetric and Gynecology Department, George Washington University Hospital, Washington, D.C. USA; University College London, Elizabeth Garrett Anderson Institute for Women’s Health, London, United Kingdom; University College London Hospital, Women’s Health Division, London, United Kingdom; NIHR University College London Hospitals Biomedical Research Centre, London, United Kingdom

Robotic surgery has revolutionised gynaecological surgery—not only have gynaecological surgeons pioneered this important technology, gynaecologists comprise of the highest volume of robotic surgeons (Moglia, 2018). The benefits of robotic surgery are extensive and its features enable and amplify the surgeon’s skills, facilitating minimally invasive approaches for patients that would have otherwise required laparotomy (Liu et al., 2012; Peters et al., 2018). Robotic surgery requires new skills to be mastered by gynaecological surgeons and trainees. It demands a different finesse and expertise from conventional laparoscopy and laparotomy. Although robotic skills have a smaller learning curve when compared to traditional laparoscopy, elemental and foundational knowledge about the robotic platform are critical to learn prior to a live case in order to perform robotic surgery safely and effectively (Kowalewski et al., 2018; Stefanidis et al., 2010). Prior research shows that learners who have undergone a formal robotics curriculum gain proficiency in a shorter prior of time when compared to learners without structured training (Rocha et al., 2016; Van Der Poel et al., 2016; Volpe et al., 2015). These curriculums have used a variety of implementation platforms, including web-based didactics, on-site training programmes, and simulation (Arain et al., 2012; Connolly et al., 2014; Green et al., 2018; Ismail et al., 2020). Unfortunately, there is a lack of consistency and standardisation across these various modalities as individual programmes have different requirements and their certification protocols vary widely (Chen et al., 2020; Moit et al., 2019; Tom et al., 2019; Winder et al., 2016). Currently, there is no comprehensive, universally accepted robotic curriculum to train and assess fundamental competency of surgeons for robotic surgery (Satava et al., 2020). As robotic surgery continues to expand, the creation of standardised, quality assured, certified training pathways is imperative, which include web-based training, simulation, on-site training, and mentorship. In this editorial, we aim to highlight the various features of existing curricula and propose a guideline on how to best merge these processes to create a universal protocol for robotic surgery training.

## Current platforms for robotic curricula

### Web-Based Training

This learning modality is completely virtual. The online didactics coach novices through educational knowledge using interactive modules and full-length procedure videos. The most widely incorporated programmes include the Fundamentals of Robotics (FRS), Medtronic’s Hugo, CMR Surgical’s Versius, and Intuitive’s DaVinci Technology Training. These curricula cover important critical knowledge for robotics procedures during the pre-operative, intra-operative, and post-operative phases. The didactics review appropriate patient selection, patient and system positioning, port placement, machine trouble shooting, device functions, instrumentation, etc. A key advantage of web-based learning is flexibility; learners can choose when to partake in this virtual learning and self-schedule their didactics. Additionally, trainees can easily review modules, allowing better retention of this important information. However, a disadvantage of completely online didactics is the absence of active mentorship. While videos may help lay a foundation for robotic procedural skills, they do not provide real-time performance feedback. Without active feedback, trainees can potentially ingrain inefficient motor patterns and thought processes that can negatively impact their surgical skill acquisition.

### Simulators

Web-based training programmes offer limited robotic console experience, and accurate utilisation of robotic console is a requisite to achieve surgical proficiency (Foote and Valea, 2016). Additionally, simulation training allows a safe practice space for trainees, prior to a procedure on a patient (Kneebone, 2009). Traditionally, after completion of the online didactics, learners begin practicing on a simulator. A multitude of simulator consoles exist; essentially, they provide learners with a chance to familiarise themselves with the surgeon console and skills such as energy application, needle control, etc (Kenney et al., 2009; Schreuder et al., 2014; Yang et al., 2017). Some simulators are equipped with procedure specific 3D case videos to improve clinical decision-making and procedural knowledge. The literature supports the validity of surgical robotic simulators in teaching essential robotic skills and reducing the initial learning curve of robotic surgery (Chen et al., 2020). Despite increased application of robotic surgery worldwide, training with simulators is not widely available to learners. Cost is a factor for access to robotic simulators, especially in rural areas and in hospitals with limited resources. Simulators are an important stepstone in robotic surgery proficiency, as completion of the simulation modules is a milestone to achieve prior to sitting at the console for the first time in a live case (Chowriappa et al., 2015, 2013).

### On-site training

If simulation is not accessible, or in addition to simulation training, on-site hands-on exposure can occur at dedicated training facilities. Multiple surgical organisations worldwide host conferences specifically for robotic surgery (notably SAGES, Society of American Gastrointestinal and Endoscopic Surgeons; American Association of Gynecologic Laparoscopists, AAGL; Society of European Robotic Gynaecological Surgery, SERGS; and Intuitive, the makers of the DaVinci surgical robot) (Chen et al., 2020). These tend to be intensive surgical ‘boot camps’ to help learners, after completion of online didactics, practice psychomotor skills and surgical procedures in the presence of expert robotic surgeons and trainers. The corrective live feedback that was absent from web-based and simulator platform is present in these on-site training sessions. Learners can develop and ingrain efficient motor patterns and skills for robotic surgery with active participation at these sessions (Jones et al., 2017; Siddiqui et al., 2014). Analogous to the simulator platform, accessibility and cost present as significant barriers to attendance of these on-site training programmes. Additionally, if simulators are not available at the learner’s home base, maintenance of learned skills from the on-site sessions is challenging.

### Mentorship- Precepting and Proctoring

After familiarity with equipment and simulation experience, the next steps are preceptorship and proctorship. Preceptors antecede proctors. A preceptor is a surgeon who is solely responsible for the patient and can complete the entire robotic case. This preceptor guides the trainees through robotic surgery, giving feedback on technique and skill during and after the procedure. This is a very different role from a proctor. A proctor is an experienced robotic surgeon who is evaluating a surgeon’s competence as he or she completes a robotic surgery. Proctors do not perform any part of the operation. Preceptorship is required for most robotic credentialing, but proctorship requirements vary in hospitals around the world (Green et al., 2020). Proctorship requires in person evaluation, unfortunately, some novice robotic surgeons have limited access to this proper mentorship given cost and location. However, social media has allowed non-traditional mentorship and engagement opportunities, and it has the potential to fill this proctorship void. Novice surgeons are able to upload video clips to social media groups for feedback from mentors (Jones et al., 2017). Additionally, robotic consoles have the ability for tele-mentoring. An off- site expert surgeon can communicate with the surgeon and operating room team in real time from a remote location to offer live feedback and guidance (Chen and Falcone, 2009). Telesurgery could be important tool for future robotic surgery credentialing.

## Establishing a standardised robotic curriculum

Previous research has established the importance of having a curriculum for robotic surgery as it improves learner efficiency and clinical outcomes; we also know that the various components of the previously mentioned curricula have a beneficial component (Chen et al., 2020; Larcher et al., 2019; Rusch et al., n.d.; Satava et al., 2020). While the authors acknowledge that creating a united global curriculum would be laborious, we believe robotic surgical training must be standardised in order for the continued growth and implementation of robotic surgery and the maintenance of excellent patient care and outcomes. Key questions to consider in the development of this curriculum include:

- Who will be the accrediting body?
- How do we validate this new global curriculum? Do we have different curriculums for residents, fellows, or experienced surgeons?
- How do we ensure feasibility and consistency in this curriculum?
- How do we involve different manufacturers and ensure the curriculum is applicable to all available systems?
- Who provides the funding and how can we keep the training affordable?
- What would robotic certification maintenance entail and re-certification?

While these questions are difficult to answer immediately, steps need to be taken to help reach a consensus. The research supports standardisation of surgical curriculum, and while constructing a universally accepted robotic curriculum will be arduous, it is an instrumental step to propel robotic surgery forward.

Complex topics in curricula development: In this section, we will address some the important components and concerns in designing a worldwide robotic curriculum. First, what components of the available curriculum platforms should be included in the universal robotic curriculum? In addition, how do we define competence in these components? The authors of this article feel implementation of uniform web-based modules as the initial portion of the curriculum is an important, achievable component that is both low cost and easily available to all learners. Each module should be accompanied by a quiz, in which the learner must score highly (i.e. 80% or higher) to move onto the next module. A final quiz should be administered on completion of the entire web-based curriculum. Only with a passing certificate should the trainee be able to move on to the next step of the curricula. The established literature clearly demonstrates the importance of simulation in surgical training. Similar to the web-based component, all simulation modules must have an 80% pass rate and must be completed prior to the next step, the surgeon console. However, we previously mentioned that not all trainees have access to a robot simulator. If a learner does not have access, the accrediting body can help navigate access to robotics simulators at on-site training programmes. On-site training can be optional for an additional cost if learners with access to simulators also would like additional supervised training on a robotic trainer. Therefore, completion of the required simulation modules and subsequent certification can be done on at the home institution or at an on-site training programme. After completion of both web-based and simulation modules, the next step is mentorship. Robotic mentors need to have been robotic certified by the accrediting body and trained on how to precept and/or proctor. The definition of high volume surgeon varies by surgical specialty, but commonly at least procedures more than once a month (Mowat et al., 2016). From this number, we can extrapolate a reasonable number of at least 12 bedside assists and 12 preceptorships before proctorship can begin. An additional 10 surgeries with proctorship should be required to successfully complete the entire robotic curriculum. Another important discussion point includes the differing robotic platforms; the expiration of Intuitive’s DaVinci robot patents has allowed other companies to launch their own robotic surgical machines into the market, such as Avatera, Hugo, Versius, etc (Koukourikis and Rha, 2021). While different robotic devices are important for new innovations, surgical outcomes, and patient safety, this poses a problem for standardising a curriculum. Additionally, it is imperative that the governing body of the universal curriculum does not endorse a particular robotic company, as this stalls innovation and creates an unfair monopoly. However, this means the universal curriculum must be adaptable to the available robotic platforms. Many principles of robotic surgery remain the same, such as establishing initial laparoscopic access, management of gas, principals of electrosurgery, etc. However, there are particulars to each robotic system, for example trocar placement, robot docking, available tools, haptic feedback, etc. The web-based modules, simulation/on-site training, and mentorship must be adjustable to these specific robotic platforms. Robotic surgery is important to a multitude of specialties (gynaecology, colorectal, urology, etc), so one must ask, when should the global curriculum allow for divergence into these specialties? Luckily, many robotic simulators already have specialty-related simulation as part of the modules. These can easily be incorporated into the required simulation modules. For example, a gynaecology-learner would be required to do a simulated hysterectomy on a robotic simulator. Another key portion for specialty refinement would be during the mentorship phase, as those fields are when the learners will be operating with guidance in their particular field.

Prior meetings about robotic curriculum: In 2020, the Orsi Consensus Meeting on European Robotic Training (OCERT) established an expert panel to discuss the modernisation and standardisation of robotic surgery (Vanlander et al., 2020). Similar studies and committees in the United States have come to the same conclusion (Chen et al., 2020). We need to bring robotic surgical specialties together for input and consensus on a global robotic curriculum. It is clear to address the growing demand for competent robotic surgeons, we need to facilitate the development of a standardized robotic surgical curriculum for education and credentialing.

In conclusion, implementation of a structured worldwide robotic curriculum is recommended. Regulation and monitoring of robotic training is necessary to adequately train robotic surgeons and ensure patient safety.
